# A key role for the exoribonuclease XRN1 in regulating the hepatitis B viral transcriptome

**DOI:** 10.1016/j.isci.2026.116328

**Published:** 2026-06-11

**Authors:** Senko Tsukuda, Nadina Wand, James M. Harris, Olivia Dobrica, Medhi Boutasbih, Xiaxuan Zhu, Peter A.C. Wing, Peter Balfe, Jane A. McKeating

**Affiliations:** 1 Chinese Academy of Medical Sciences Oxford Institute, University of Oxford, Oxford, UK; 2 Nuffield Department of Medicine, University of Oxford, Oxford, UK

**Keywords:** Molecular biology, Virology

## Abstract

Chronic hepatitis B virus (HBV) is a leading cause of liver disease and hepatocellular carcinoma that persists as a DNA mini-chromosome and replicates via genomic RNA intermediates. While HBV replication has been extensively studied, mechanisms governing viral RNA degradation remain unclear. Here, we identify a key role for the 5′–3′ exoribonuclease XRN1 in regulating the HBV transcriptome. Pharmacologic inhibition or genetic ablation of XRN1 increased HBV subgenomic RNAs without affecting pregenomic RNA (pgRNA) levels. Long-read RNA-seq showed that subgenomic RNAs, but not pgRNA, are degraded by XRN1, consistent with differential 5′ cap modification and processing (P-) body localization. HBV infection increased P-body number and size, and delivery of synthetic pgRNA demonstrated that active replication, not RNA expression alone, alters the abundance of P-bodies. These findings identify XRN1 as a key regulator of HBV RNA turnover and uncover a mechanism for viral replication to remodel host RNA dynamics.

## Highlights

• XRN1 regulates both the abundance of P-bodies and their size
• XRN1 regulates the HBV transcriptome via its enzymic activity
• Localization of HBV subgenomic RNA to P-bodies correlates with its degradation
• HBV replication alters the abundance of P-bodies

## Graphical abstract

**Figure:**
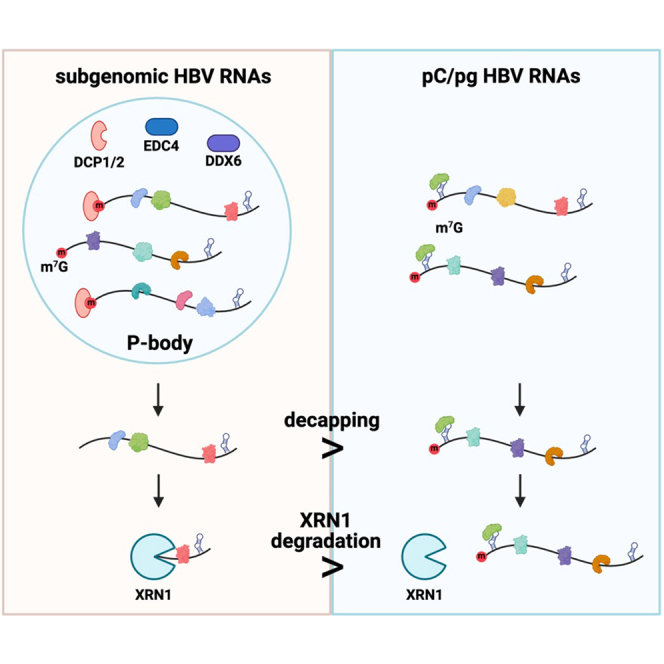

## Introduction

Hepatitis B virus (HBV) is a small 3.2 kb DNA virus and a prototypic member of the *hepadnaviridae* family that causes acute or chronic hepatitis. Chronic hepatitis B (CHB) is a global health problem, with 2 billion people exposed to the virus during their lifetime, resulting in ∼250 million chronic infections. CHB associates with an increased risk of progressive liver disease, including cirrhosis and hepatocellular carcinoma, with an estimated 820,000 deaths annually.^1^ HBV infects hepatocytes through the sodium taurocholate co-transporting polypeptide (NTCP) receptor^2^^,^^3^ and persists in the nucleus as a covalently closed circular DNA (cccDNA) complexed with histones. This cccDNA is transcribed by host RNA Polymerase II to yield six major overlapping transcripts that are 5′-capped and share a 3′ polyadenylation signal: pre-core (pC) that encodes e antigen (HBeAg); the bicistronic pre-genomic RNA (pgRNA) that can yield core protein (HBc) and polymerase; the preS1, preS2 and S RNAs that encode the surface glycoproteins (HBs) and X transcript that encodes the regulatory HBx protein.^4^ Our recent long-read RNA sequencing studies show that transcripts encoding HBs dominate the viral transcriptome, while the genomic-length pC and pgRNA represent a relatively minor component (∼8%).^5^^,^^6^ pgRNA is unique among these transcripts in its ability to assemble into capsids, a process involving the 5′ epsilon (ε) stem-loop and other *cis*-elements, where polymerase-mediated reverse transcription generates new infectious particles.^7^

Current nucleoside analog therapies rarely cure infection as they have limited impact on the long-lived HBV cccDNA that constitutes the viral reservoir.^8^ Several new therapeutic strategies, including HBV-specific small interfering RNAs (siRNAs) and epigenetic modifying agents, are in development with the goal to cure infection.^9^^,^^10^^,^^11^ Although the mechanisms of HBV replication have been well studied, comparatively little is known about the cellular pathways responsible for degrading viral RNAs. The 5′–3′ RNA degradation machinery (5-3DM) mediates mRNA decay and comprises the exonuclease XRN1, decapping proteins DCP1 and DCP2, the enhancer of mRNA-decapping protein (EDC4), and several accessory cofactors.^12^ RNA decay is recogniZed as a mechanism for remodeling the transcriptome in response to cellular stress.^13^^,^^14^ XRN1 and other 5-3DM components localize to phase-separated, membraneless organelles known as processing (P-) bodies: Dynamic structures formed via networks of protein-protein, protein-RNA and RNA-RNA interactions that are conserved amongst all eukaryotes.^15^^,^^16^ More recently, P-bodies have been identified as storage hubs for microRNAs and translationally repressed mRNAs.^17^^,^^18^^,^^19^

XRN1 is known to target several mammalian pathogenic RNA viruses (Newcastle disease virus, Encephalomyocarditis virus, Sendai virus, Coxsackievirus B).^20^^,^^21^^,^^22^^,^^23^^,^^24^ Many of these viruses have evolved mechanisms to evade XRN1: For example, poliovirus induces the proteasomal degradation of XRN1,^25^ while flaviviruses encode pseudoknot RNA structures in their 3′ untranslated region that inhibit XRN1 activity, producing XRN1-resistant RNAs (xrRNAs) that can suppress the host innate response.^21^^,^^22^^,^^23^^,^^24^ In contrast, DNA viruses are transcribed by the host machinery and produce transcripts modified with a 5' N7-monomethylated guanosine (m^7^G) cap structure; these transcripts are generally assumed to resist XRN1-mediated degradation. Recent studies from the cancer literature highlight a surveillance role for XRN1 in degrading endogenous retroviral RNAs that are frequently activated in tumors.^26^^,^^27^^,^^28^ XRN1 degrades the long non-coding RNAs that regulate cell differentiation^29^ and promote the turnover of tumor-suppressive microRNAs.^30^ Collectively, these studies support a role for XRN1 in regulating RNA homeostasis.

Here, we investigate the role of the 5′-3′ exonuclease XRN1 in HBV infection using pharmacological inhibition, XRN1 knockout (KO), and overexpression approaches. We show that sub-genomic HBV RNAs localize to P-bodies and can be degraded by XRN1, whereas pgRNA is resistant.

## Results

### XRN1 degrades HBV RNAs

**Figure 1:**
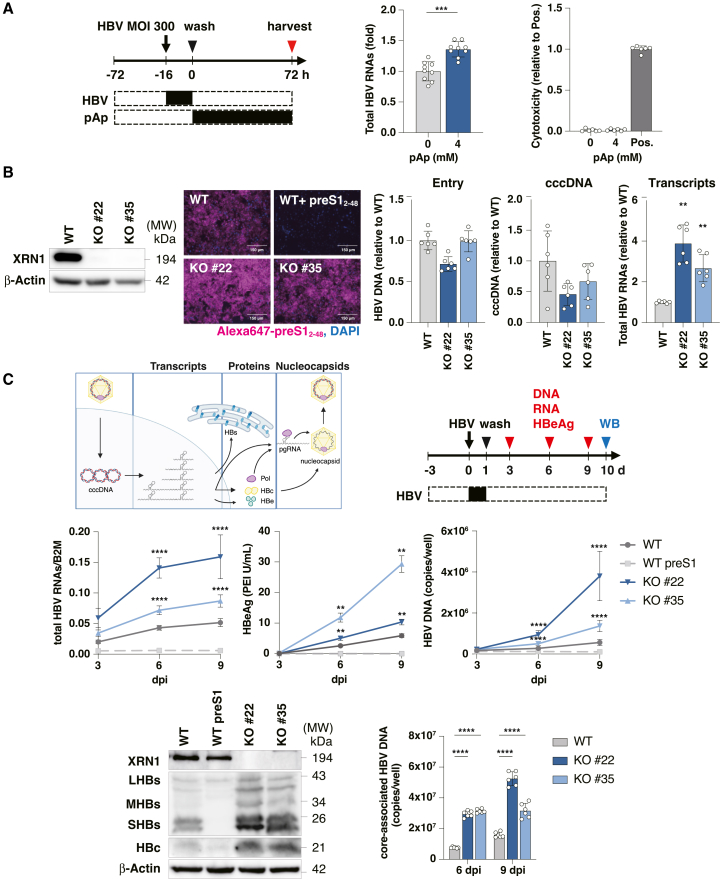
XRN1 limits HBV RNAs (A) HBV infection and pAp treatment. Arrested HepG2-NTCP cells were infected with HBV for 16 h, inoculum removed, and cells treated with vehicle or 4 mM 3′, 5′-bisphosphate (pAp) for 72 h. RNA was extracted and total HBV RNAs quantified by RT-qPCR, normalized to the housekeeping gene *B2M* and expressed relative to vehicle-treated cells (mean ± SD, *n* = 9 from 3 independent experiments, one-way ANOVA, with multiple comparisons, ∗∗∗*p* < 0.001). Cytotoxicity was evaluated by LDH assay with data expressed relative to cells treated with 0.05% Triton X-100 (Pos). (B) HBV infection of WT and XRN1 KO cells. WT and KO HepG2-NTCP cells were assessed for XRN1 expression by western blotting and treated with 50 nM Alexa 647 conjugated preS1_2-48_ peptide in the presence or absence of excess unlabeled peptide (200 nM). After washing, the cells were incubated with DAPI and visualized by microscopy with a 20*×* objective, scale bars, 150 μm. WT and XRN1 KO HepG2-NTCP cells were inoculated with HBV for 16 h. Intracellular DNA was extracted after 16 h post-infection and RNA at 72 h post-infection. HBV DNA, cccDNA, and total HBV RNAs were quantified by qPCR and normalized to the housekeeping genes, *PrP or B2M*, respectively. Data are plotted relative to the WT cells (mean ± SD, *n* = 6 from 2 independent experiments). (C) HBV life cycle and kinetic analysis of infection. WT and XRN1 KO HepG2-NTCP cells were inoculated with HBV for 16 h in the presence or absence of preS1_2-48_ peptide, unbound virus removed by washing, and the cells cultured for 3, 6, 9, or 10 days. The schematic depicts steps in the HBV life cycle that were evaluated. Intracellular HBV RNA and secreted HBeAg were quantified at 3, 6, and 9 dpi by RT-qPCR and ELISA, respectively. RT-qPCR data are expressed relative to the housekeeping gene, *B2M* (mean ± SD, *n* = 9 from 3 independent experiments). Intracellular rcDNA and core-associated HBV DNA were quantified by qPCR (mean ± SD, *n* = 6 from 3 independent experiments). XRN1, HBs, HBc, and β-Actin proteins were detected at 10 dpi. Statistical significance was determined using Mann-Whitney tests, with Bonferroni correction for multiple comparisons; ∗∗*p* < 0.01, ∗∗∗*p* < 0.005, and ∗∗∗∗*p* < 0.0001. Please see also Figure S1.

To assess whether HBV RNAs are sensitive to XRN1, we treated infected HepG2-NTCP cells with the XRN1 inhibitor 3′,5′-bisphosphate (pAp)^31^ and quantified viral RNAs by RT-qPCR using primers targeting the 3′ region shared by all transcripts. Treatment induced a modest but significant increase in viral RNA levels, with minimal evidence of cytotoxicity (Figure 1A). To extend these observations, we generated XRN1 KO HepG2-NTCP cells and identified two lines (22 and 35) that proliferated at a similar rate to the parental wild-type (WT) cells (Figures 1B and S1). Both KO lines expressed NTCP and supported comparable HBV entry and cccDNA levels to WT cells (Figure 1B). We noted a significant increase in the steady state levels of viral transcripts in both KO cells (Figure 1B). To assess the wider impact of the increase in viral RNAs on the HBV life cycle, we measured viral-encoded proteins and nucleocapsids in WT, KO22, and KO35 cells at 3, 6, and 9 days post-infection (dpi) (Figure 1C). To ensure these markers represent *de novo* viral replication events, we pre-treated the cells with a myristoylated peptide encoding amino acids 2–48 of the large surface glycoprotein (preS1_2-48_) that binds NTCP and neutralizes viral infection. We observed a time-dependent increase in viral RNA, HBeAg, and intracellular HBV DNA expression, with significantly higher levels in the KO22 and KO35 cells (Figure 1C). HBc and HBs glycoproteins play a key role in the envelopment of capsids, and we noted an increase in their expression in the KO cells, consistent with elevated levels of core-associated HBV DNA (Figure 1C).

**Figure 2:**
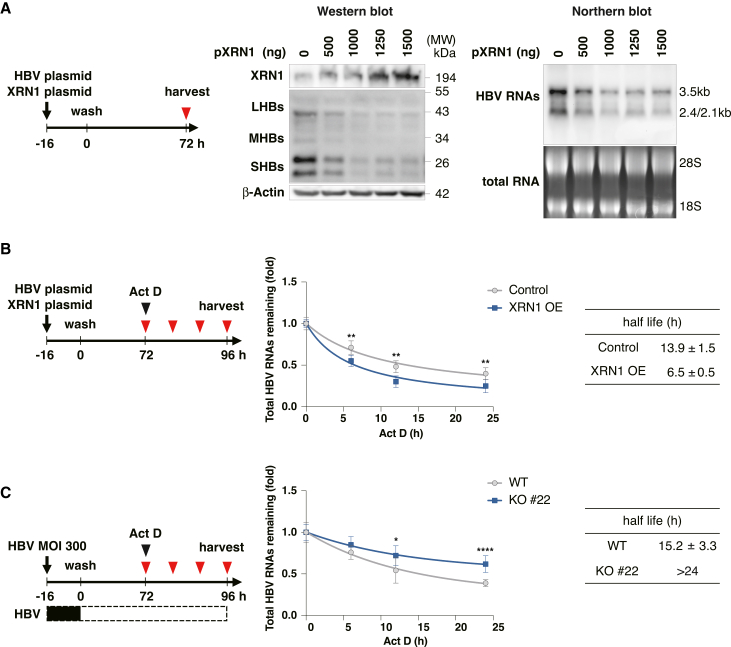
XRN1 modulates HBV RNA abundance and half-life (A) HBV protein and RNA levels in XRN1 overexpression. HepG2-NTCP cells were transfected with a transcriptionally competent HBV 1.3*×* overlength plasmid together with the increasing concentration of an empty vector (control) or an XRN1 expression plasmid for 72 h. XRN1, HBs, and β-Actin were detected by western blotting and viral RNAs by northern blotting, with 18S and 28S rRNAs detected as a loading control. (B and C) XRN1 perturbs HBV RNA half-life. HepG2-NTCP WT cells were co-transfected with 50 ng HBV 1.3*×* overlength plasmid together with 250 ng XRN1 or control plasmids (B). HepG2-NTCP WT or XRN1 KO22 cells were infected with HBV (C). Cells were treated with actinomycin D (Act D) and harvested after 0, 6, 12, or 24 h. Total HBV RNA levels were quantified by RT-qPCR, and the data presented relative to the 0 h Act D treatment, where the plot presents the mean ± SD of *n* = 6 or 9 replicates from three independent experiments. Viral RNA half-life was estimated by nonlinear regression (one-phase exponential decay) in 3 independent experiments. Statistical significance was determined using Mann-Whitney tests, with Bonferroni correction for multiple comparisons; ∗*p* < 0.05, ∗∗*p* < 0.01, and ∗∗∗∗*p* < 0.0001. Please see also Figures S2 and S3.

To complement the KO experiments, we over-expressed XRN1 by co-transfecting plasmids encoding the exonuclease with a transcriptionally active HBV genome. Our earlier study comparing the viral transcriptome following HBV plasmid transfection or virus infection of HepG2-NTCP cells showed a comparable pattern of viral RNAs,^6^ validating this approach. We observed a dose-dependent increase in XRN1 protein expression that associated with a reduced intensity of genomic-length pC/pgRNAs (3.5 kb) and sub-genomic preS1/preS2/S transcripts (2.4/2.1 kb) (Figure 2A). Longer exposure of the northern blots showed limited evidence for degraded RNAs in the XRN1 over-expressing cells, most likely reflecting the rapid digestion rates reported for this exonuclease^32^ (Figure S2A). Consistent with the XRN1-driven degradation of preS1/preS2 and S RNAs, we noted the reduced expression of all HBs isoforms, HBeAg, and HBV DNA (Figures 2A and S2B). To estimate the half-life of the total HBV RNAs in the XRN1 overexpressing cells and the infected XRN1 KO22 line, we treated the cells with actinomycin D (Act D) and quantified viral RNAs by RT-qPCR. Overexpression of XRN1 reduced their estimated half-life to 6.5 ± 0.5 h compared to 13.9 ± 1.5 h in the control cells (Figure 2B). In line with these results, we observed a significant increase in the stability of the HBV RNAs to >24 h in the infected XRN1 KO cells, compared to 15.2 ± 3.3 h in the WT cells (Figure 2C). To determine whether XRN1 targets host factors reported to suppress or activate HBV transcription (reviewed in^33^) we sequenced RNA from WT and KO22 cells. There was a modest increase in the gene expression of Krüppel-like factor 15 and a reduction in Fos Proto-Oncogene gene; however, neither was significant (Figure S3). In summary, these complementary approaches show a role for XRN1 in the degradation of HBV RNAs.

### Degradation of genomic and sub-genomic HBV RNAs

**Figure 3:**
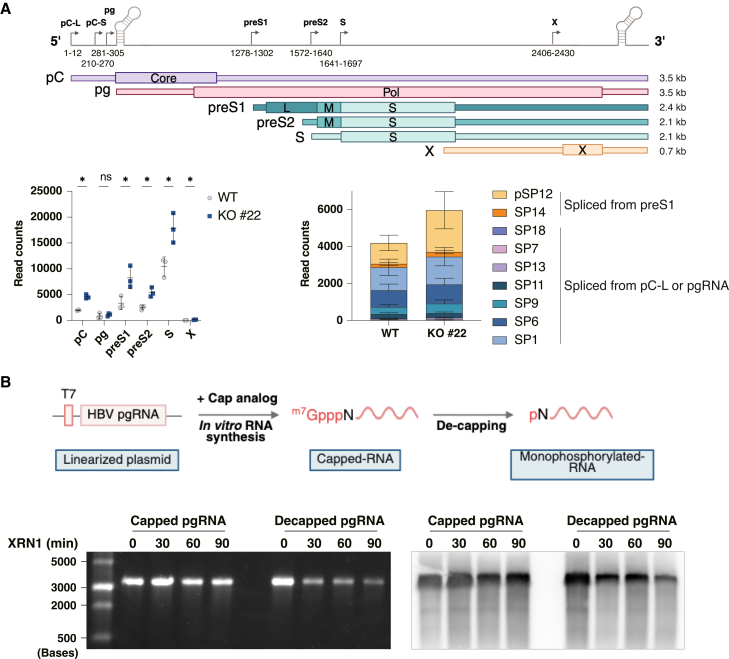
XRN1 regulation of the HBV transcriptome (A) HBV transcript mapping. Cartoon depicting the genomic viral RNA with the 5′ and 3′ epsilon stem loops, canonical transcription start sites, and major viral RNAs along with open reading frames (core, Pol, L, M, S, and X). HepG2-NTCP WT or XRN1 KO22 cells were infected with HBV, RNAs extracted at 6 dpi, and analyzed by long-read sequencing. Read counts for pC, pg, preS1, preS2, S, and X transcripts (left), along with spliced isoforms (right), are shown. Data are presented for 6 independent samples and statistical significance assessed using unpaired t tests (corrected for multiple comparisons, ∗*p* < 0.05). (B) *In vitro* pgRNA digestion by XRN1. Capped-analog modified mRNA was synthesized by *in vitro* transcription. The capped and decapped pgRNA was incubated with XRN1, evaluated by agarose gel electrophoresis, and northern blotting using an HBV probe. Please see also Figure S4.

To determine whether all HBV RNAs are equally sensitive to XRN1, we sequenced the viral transcriptome from infected WT and KO22 cells at 6 dpi using a recently published targeted long-read method.^6^ Sequences were demultiplexed to generate libraries and aligned to a linearised HBV D3 genome (HBV genotype D, GenBank accession no. X02496.1) using minimap2 with transcripts identified using reported transcription start and termination sites.^34^^,^^35^ The preS1, preS2, and S transcripts constituted ∼75% of the reads, with lower levels of genomic-length pC and pg along with X RNAs, consistent with our earlier reports.^5^^,^^20^ The poly(A) tail lengths of all viral transcripts were longer in the KO22 cells (Figure S4A), and this may reflect a pleiotropic role for XRN1 in regulating RNA metabolic pathways (Figure S4B). The majority of canonical and spliced mRNAs were significantly reduced in the WT compared to the KO22 cells, consistent with their degradation by XRN1 (Figure 3A). In contrast, pgRNA levels were similar, suggesting a resistance to XRN1 exonuclease activity.

Ruscica et al.^36^ reported that XRN1-resistant mRNAs showed altered dinucleotide frequencies (notably CpG and ApG).^36^ Quantifying the dinucleotide frequencies in the HBV mRNAs showed a similar pattern of CpG usage in all transcripts, suggesting this mechanism does not explain pgRNA resistance to XRN1 degradation. We hypothesized that the ε sequence within the 5′ pgRNA forms a structured hairpin motif.^37^^,^^38^ which may block 5′-3′ exonuclease activity. To evaluate this, we incubated *in vitro* transcribed pgRNA with recombinant XRN1 in a cell-free assay. We observed a time-dependent degradation that was m^7^G-cap dependent (Figure 3B), showing a minimal protective role for the ε sequence *per se*. This conclusion is supported by the observation that the ε-containing pC transcripts were more abundant in the KO22 cells and are targets for XRN1-degradation. Collectively, these data show a key role for XRN1 in the regulation of HBV sub-genomic RNAs and highlight the resistance of pgRNA.

### HBV RNAs co-localize with the P-body components EDC4 and DDX6

**Figure 4:**
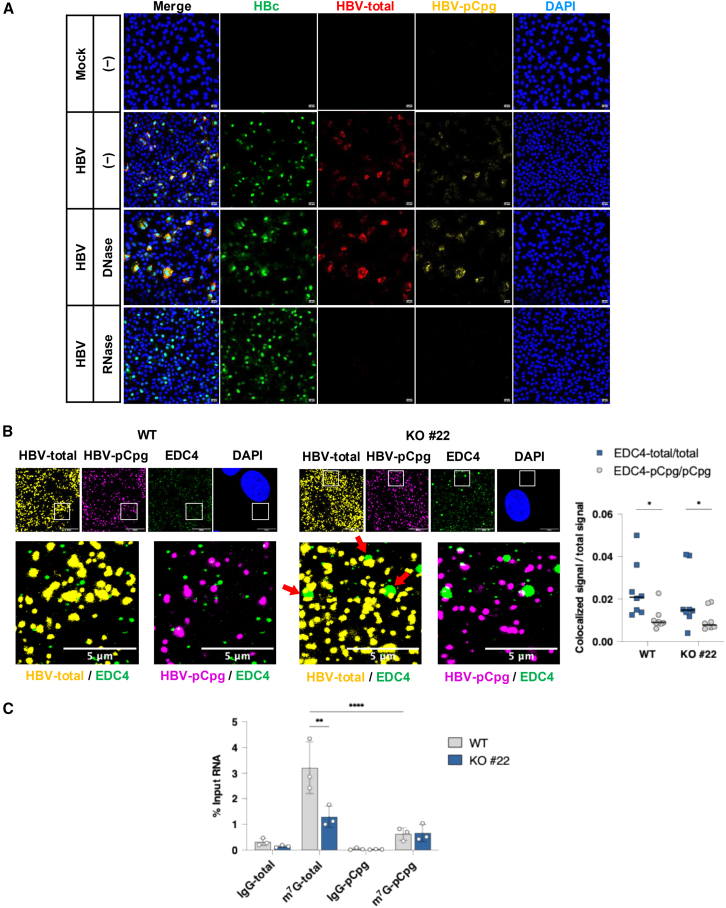
XRN1 regulates P-body formation and capped viral RNAs (A) FISH of HBV RNA. Mock or HBV-infected HepG2-NTCP cells (6 dpi) were treated with DNase I or RNase T/RNase A/RNase III, prior to hybridization with HCR probes targeting HBV-total or HBV-pC/pg RNA together and stained for HBc expression by conventional antibody-based immunofluorescence (scale bars, 20 μm). (B) Colocalization of HBV RNAs with EDC4 puncta. HBV-infected HepG2-NTCP WT or KO22 cells (6 dpi) were imaged for HBV-total (yellow), HBV-pC/pg (magenta), EDC4 (green), and nuclear DAPI (blue), with scale bars of 10 μm (main image) and 5 μm (inset). Red arrows highlight colocalized HBV RNA-EDC4 large puncta that were a notable feature of the KO22 cells. The frequency of HBV-total or HBV-pC/pg RNA puncta colocalizing with EDC4 was quantified from 8 images collected from two independent infections, and the average was expressed relative to the overall viral signal, with significance assessed using Mann-Whitney tests (∗*p* < 0.05). (C) Quantification of capped HBV RNA. Total cellular RNA from infected HepG2-NTCP WT or KO22 cells at 6 dpi was immunoprecipitated with anti-m^7^G or an irrelevant IgG, and viral RNAs were measured by RT-qPCR. The data are presented as the mean ± SD of 3 independent experiments and significance assessed using a Mann-Whitney test (∗∗*p* < 0.01). Please see also Figures S5–S7.

To assess the sub-cellular localization of viral transcripts and their proximity to P-bodies, we developed fluorescent *in situ* hybridization (FISH) probes targeting pC and pg RNA (HBV-pCpg) or all RNAs (HBV-total) (Figure S5) and realized their signal using hybridization chain reaction (HCR). HBV HCR signals were ablated by RNase digestion pre-hybridization, confirming the specificity of the probes. Furthermore, DNase digestion prior to hybridization in HBV infected cells had no impact on the HCR signal pattern or intensity, confirming the probes did not cross react with HBV DNA (Figure 4A). The concordant detection of cells expressing both viral RNA and HBc antigen confirmed probe specificity (Figure 4A). Our attempts to image XRN1 using several commercially available antibodies resulted in non-specific staining, and we therefore imaged EDC4, a component of P-bodies, that interacts with XRN1.^19^ High-resolution imaging revealed abundant small EDC4 puncta in both WT and KO infected cells, with noticeably larger puncta (indicated by red arrows) in KO22 cells (Figure 4B). Similar increases in the frequency and size of EDC4 puncta were observed in the uninfected KO22 cells (Figure S6). HBV-total RNAs co-localized with EDC4 puncta at a significantly higher frequency than HBV-pC/pg in the WT and KO cells (Figure 4B), suggesting that P-body condensates sequester viral RNA. To confirm these observations, we showed that total HBV RNAs co-localized with a second P-body component, DDX6 (Figure S7).

As EDC4 is integral to mRNA decapping, we assessed whether there was differential capping of the viral RNAs using an antibody targeting the m^7^G cap to precipitate RNA from infected WT and KO22 cells. The relative amounts of cap-modified RNAs were estimated by RT-qPCR using primers targeting the 3′ region shared by all HBV transcripts or the 5′ end of the genome to identify pC and pgRNA. We noted a significant reduction in capped total HBV RNA in the KO22 cells that reflects their higher co-localization with EDC4, whereas the amount of precipitated pC and pgRNA was similar in both cell lines (Figure 4C). The preferential localization of HBV-total over HBV-pCpg to P-bodies suggests a mechanism by which pgRNA evades decapping and XRN1-dependent degradation.

### HBV infection increases P-body number and size

**Figure 5:**
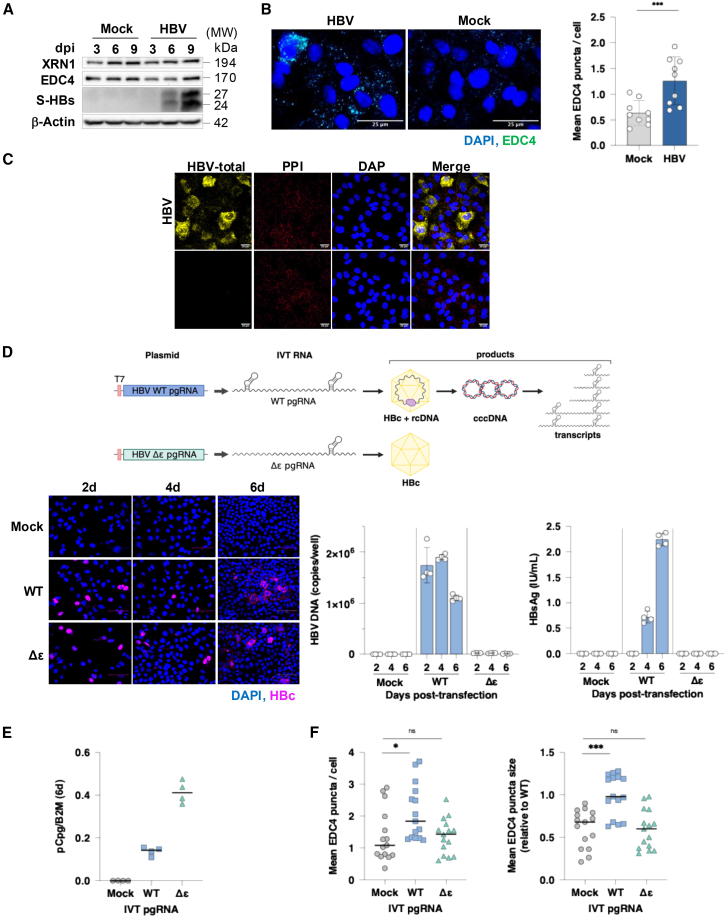
HBV induces P-bodies (A and B) Effect of HBV infection on XRN1 and EDC4 expression. Mock or HBV-infected HepG2-NTCP cells were cultured for up to 9 days (A) P-body components XRN1 and EDC4, together with HBsAg and β-actin, were detected at 3, 6, and 9 dpi by western blotting. (B) EDC4 (green) and DAPI nuclei (blue) were detected by immunofluorescence at 6 dpi, with a representative image shown (scale bars, 25 μm). The average number of EDC4 puncta relative to nuclei was quantified in 9 images from 3 independent experiments, with significance assessed using a Mann-Whitney test (∗∗∗*p* < 0.001, ∗*p* < 0.05). (C) Abundance of viral and host RNAs. Total HBV RNAs (HBV-total, yellow) and host *Peptidylprolyl isomerase B RNA* (PPIB, red) and nuclear DAPI (blue) were imaged at 6 dpi (scale bars, 20 μm). (D) Replication of *in vitro* transcribed HBV pgRNA. A schematic of *in vitro* transcribed (IVT) WT or Δε pgRNA and their viral products. Mock or IVT HBV RNA-transfected (100 ng) Huh 7.5 cells were cultured for 2, 4, or 6 days. Cells were imaged for HBc (magenta) (scale bars, 75 μm), intracellular HBV DNA, and secreted HBsAg assessed by qPCR or ELISA, respectively. (E) pCpg RNA levels after 6 days were measured by RT-qPCR, and the data were normalized to a housekeeping gene, *B2M*, respectively. (F) EDC4 puncta in HBV RNA-transfected cells. Mock or IVT HBV RNA-transfected (100 ng) Huh 7.5 cells (6 days) were stained for EDC4 expression and puncta number and size quantified in 15 images from 3 independent experiments. Mean EDC4 values are expressed relative to the number of nuclei per image, with statistical significance assessed using a Mann-Whitney test with correction for multiple comparisons (∗∗∗*p* < 0.001 and ∗*p* < 0.05).

We investigated whether HBV infection modulates P-body components. Western blot analyses of XRN1 and EDC4 in mock or infected HepG2-NTCP cells revealed little change in steady-state protein levels over the course of infection (Figure 5A). In contrast, imaging EDC4 showed a significant increase in the mean number of EDC4 puncta in the infected cultures at 6 dpi (Figure 5B). As XRN1 is a key player in 5-3DM surveillance of cellular RNA^17^ we hypothesized that a high intracellular HBV RNA burden would activate this pathway. To address this, we imaged HBV RNAs along with a housekeeper transcript *peptidylprolyl isomerase B (PPIB),* in infected HepG2-NTCP cells at 6 dpi. HBV RNA signals were abundant, with both their frequency and intensity exceeding PPIB gene expression (Figure 5C). These imaging results are consistent with high HBV gene expression that may perturb P-body dynamics. To test this hypothesis, we delivered *in vitro* transcribed pgRNA into Huh-7.5 cells using previously reported protocols.^39^^,^^40^ Transfecting WT or defective Δε pgRNA, which cannot reverse-transcribe or establish cccDNA,^39^^,^^40^ allowed us to determine whether HBV RNA alone can perturb P-bodies or if a full replicative life cycle is required (Figure 5D). Both RNAs encode HBc, with comparable frequencies of HBc-positive cells observed for both constructs. As Δε pgRNA is defective, intracellular HBV DNA and secreted HBsAg were only detected in the WT pgRNA-transfected cells (Figure 5D). At 6 days post-transfection we observed higher intracellular levels of Δε pgRNA relative to WT, consistent with limited XRN1-mediated degradation (Figure 5E). We noted a significant increase in both the number and size of EDC4 puncta in WT pgRNA expressing cells at 6 days post-transfection, whereas for Δε pgRNA these values were comparable to mock-transfected cells (Figure 5F), suggesting that the increased abundance and size of P-bodies is dependent on viral replication.

## Discussion

Understanding the host pathways that regulate HBV RNA stability is fundamental to the evaluation of curative treatments for this chronic disease. Our study demonstrates a critical role for XRN1 in the degradation of HBV sub-genomic RNAs, which influence multiple steps of the viral life cycle. Some HBV transcripts encode a 5′ stem-loop ε RNA structure analogous to flavivirus encoded xrRNAs.^21^^,^^41^ However, mapping the HBV transcriptome from WT or KO22 cells identified pgRNA as the only transcript protected from XRN1 digestion. HBV produces two genomic-length transcripts, pC and pgRNA, that encode a 5′ ε motif, but only pgRNA binds the viral polymerase to prime reverse transcription.^37^ The preferential localization to P-bodies of sub-genomic RNAs relative to genomic RNAs suggests a mechanism by which pgRNA evades decapping and XRN1-dependent degradation.

Our data support a model in which polymerase and associated host factors^38^ shield pgRNA from XRN1-mediated decay. Earlier studies showed that the spacing of the 5′ m^7^G cap and the ε structure influences polymerase binding.^42^^,^^43^ We noted that >85% of pgRNA sequences initiated at nucleotide position 296, consistent with efficient polymerase recruitment. Similar ε structures are found in the avian hepadnaviruses^44^ and the recently discovered non-enveloped fish nackednaviruses,^45^ suggesting a conserved mechanism of genome replication. There are limited structural data available on the interaction of HBV ε RNA with polymerase, with many reports focusing on the related duck HBV (DHBV). Cell-free reconstitution of recombinant DHBV ε RNA complexes identified several host co-factors required to initiate transcription.^46^ Unfortunately, synthetic HBV ε RNA-polymerase complexes failed to prime transcription, precluding biochemical studies of HBV ε RNA-host factor complexes and their role in evading XRN1-mediated degradation.^37^^,^^38^

In other virus families, RNA pseudoknots similar to the HBV ε domain stall XRN1 activity and produce truncated viral RNAs that subvert host innate immunity.^47^^,^^48^^,^^49^^,^^50^ For example, in dengue virus (DENV), these sub-genomic RNAs inhibit host dsRNA sensors.^24^ In Sindbis virus (SINV), XRN1 and the P-body component MOV10 co-localize to viral replication factories, and XRN1 KO renders cells resistant to infection.^51^ Some phleboviruses and arenaviruses employ G-quadruplex motifs to stall XRN1.^52^ Although twelve G-quadruplex motifs were identified in the HBV genome, they are found in the 5′ untranslated region of preS1 and the 3′ ε loop region and are therefore unlikely to explain the selective exonuclease resistance of pgRNA.^53^^,^^54^

Previous studies of purified P-bodies indicate that mRNAs enriched in these granules are translationally repressed, leading to the suggestion that P-bodies serve a dual role both as storage hubs and decay sites.^55^^,^^56^ Our *in vitro* studies demonstrate that HBV replication increases P-body number and size in hepatoma cells. In agreement with these observations, Perez-Vilaro et al. reported increased expression of the helicase DDX6 in liver biopsies from HBV-infected patients.^57^ HBV-infected cells showed unexpectedly high levels of viral RNAs, consistent with cellular stress and induction of P-bodies.

Many viral infections perturb P-body dynamics, with the majority of studies reporting their disruption: Poliovirus reduces P-body formation by degrading XRN1 and DCP1a, leading to enhanced replication^25^; SARS-CoV-2 nucleocapsid disrupts P-body assembly and alters the gene expression of inflammatory cytokines such as tumor necrosis factor and interleukin-6^58^; rotavirus and flaviviruses hijack components of P-bodies into virus-induced inclusion bodies that are the sites of viral RNA replication.^57^^,^^59^^,^^60^^,^^61^ In contrast, P-body assembly is induced during early stages of influenza A virus infection by the viral nucleoprotein associating with P-body components via RNA-associated protein 55.^62^ Clearly, for individual viruses, the regulation of P-bodies varies, and the precise mechanisms underpinning perturbation are poorly understood.^63^ Importantly, the majority of these reports have focused on RNA viruses that cause acute infections and suppress host RNA transcription or translation, commonly referred to as ‘host shutoff’.^64^^,^^65^ In contrast, HBV establishes a chronic infection that can persist for decades with no major impact on host gene expression. Analysis of P-body-associated RNAs has shown that up to one-fifth of cytoplasmic transcripts localize to P-bodies,^56^^,^^66^ suggesting that the HBV induction of P-bodies provides a mechanism for the viral post-transcriptional regulation of host mRNA.

### Limitations of the study

The small size of the HBV genome and its overlapping reading frames, together with low gene expression, complicates PCR-based approaches to map the viral transcriptome. Our use of probe-enriched long-read RNAseq allowed us to accurately quantify individual HBV transcripts and to identify XRN1-resistant pgRNA. Imaging both HBV RNAs and P-bodies at the single-cell level showed heterogeneity of the infected reservoir and suggests that population-based methods may not accurately model viral-host interplay, highlighting the importance of single-cell transcriptomic analyses.

## Resource availability

### Lead contact

Further information and requests for resources and reagents should be directed to and will be fulfilled by the lead contact, Jane A McKeating (jane.mckeating@ndm.ox.ac.uk).

### Materials availability

This study did not generate new, unique reagents.

### Data and code availability

• Data: All data supporting the findings of this study are found within the article and its supplemental information. The short RNA-seq data and long RNA-seq data generated and used in this study are publicly available with accession numbers documented in the key resources table.
• Code: All custom scripts and code used for the analysis in this study are publicly available on GitHub via our GitHub repository (https://github.com/TsukudaS/HBV-XRN1)
• All other Items: Any additional information required to reanalyze the data reported in this paper is available from the lead contact upon request

## Acknowledgments

Research in the McKeating laboratory is funded by the 10.13039/100010269Wellcome Trust (Investigator Award 200838/Z/16/Z and Discovery Award 225198/Z/22/Z) and the Chinese Academy of Medical Sciences (CAMS) Innovation Fund for Medical Science (CIFMS), China (grant no. 2024-I2M-2-001-1). MB is funded by a Kings College Hospital-Oxford University research agreement; XZ is funded by a CAMS training fellowship. We would like to thank: Stephan Urban (University of Heidelberg) for supplying HepG2-NTCP cells; Jochen Wettengel and Ulrike Protzer (Technical University of Munich) for purified HBV^67^; Azim Ansari (University of Oxford) for biotinylated probes; Alfredo Castello (Center for Virus Research, University of Glasgow) for XRN1 Cas9 KO reagents; Ju-Tao Guo (Blumberg Institute, USA) for HBV pgRNA launch reagents and Badran Elshenawy for advice on RNA-seq analysis.

## Author contributions

S.T. designed and conducted experiments and co-wrote the MS; N.W. designed and conducted experiments; J.M.H. designed and conducted experiments; O.D. designed and conducted experiments; M.B. provided technical support; X.Z. analyzed data; P.A.C.W. provided technical support: P.B. analyzed data and co-wrote the MS; J.A.M. designed the study and co-wrote the MS.

## Declaration of interests

The authors declare no competing interests.

## STAR★Methods

### Key resources table

**Table:** 

REAGENT or RESOURCE	SOURCE	IDENTIFIER
**Antibodies**		

Rabbit-*anti*-EDC4 (RCD8)	FORTIS	Cat# A300-745A-T; RRID:AB_2853734
Rabbit-*anti*-DDX6	FORTIS	Cat# A300-461A; RRID:AB_2856332
Horse-*anti*-HBsAg	Abcam	Cat# ab9193
Rabbit-*anti*-HBcAg	Dako	Cat# B0586; RRID:AB_2335704
Rabbit-*anti*-HBcAg	Beacle Inc.	Cat# BCL-ABPC-01
Rabbit-*anti*-XRN1	Thermo Fisher	Cat# A300-443A; RRID:AB_2606775
Mouse-*anti*-β-Actin	Sigma	Cat# A5441; RRID:AB_476744
Mouse-*anti*-7-methylguanosine (m7G)-Cap mAb	Caltag Medsystems	Cat# RN016M; RRID:AB_2921296

**Bacterial and virus strains**		

HBV	Kind Gift from Prof. Ulrike Protzer (Wettengel et al.^67^)	N/A

**Chemicals, peptides, and recombinant proteins**		

preS1_2-48_	Kind gift from Prof. Stephan Urban	N/A
Alexa 647-labelled preS1_2-48_	This paper	N/A
mRNA Decapping Enzyme	New England Biolabs	Cat# M0608S
XRN1	New England Biolabs	Cat# M0338S

**Critical commercial assays**		

HiScribe T7 High Yield RNA Synthesis kit	New England Biolabs	Cat# E2040S
HBeAg ELISA	Autobio	Cat# CL0312-2
HBsAg ELISA	Autobio	Cat# CL0310-2
CytoTox 96® Non-Radioactive Cytotoxicity Assay	Promega	Cat# G1780
HCR™ Gold RNA-FISH Kit	Molecular Instruments	https://www.molecularinstruments.com/

**Deposited data**		

Source data for all figures	Mendeley Data	https://doi.org/10.17632/3wxt7kzvpb.1
Long-read sequencing	(Harris et al.^5^)	NCBI SAR: PRJNA1227884
Short-read sequencing	This paper	NCBI SAR: PRJNA1228332

**Experimental models: Cell lines**		

HepG2-NTCP	Kind gift from Prof. Stephan Urban	HepG2-NTCP
HepG2-NTCP XRN1 KO22	This paper	XRN1 KO22
HepG2-NTCP XRN1 KO35	This paper	XRN1 KO35
Huh7.5	Kind gift from Prof. Peter Simmonds	Huh7.5

**Oligonucleotides**		

XRN1 crRNA	Kind gift from Prof. Alfredo Castello	N/A
Primers	See Table S1	N/A
HCR probes for HBV pC/pgRNA	See Table S2	N/A
HCR probes for total HBVs	See Table S3	N/A
HCR probes for PPIB	See Table S4	N/A

**Recombinant DNA**		

pLNHA-C1-HsXRN1	(Braun^68^)	pLNHA-C1-HsXRN1
HBV x1.3 plasmid	Kind gift from Prof. Aleem siddiqui	N/A
pUC59-pgRNA WT	Kind gift from Prof. Ju-Tao Guo (Zhao^40^)	N/A
pUC59-pgRNA Δε	Kind gift from Prof. Ju-Tao Guo (Zhao^40^)	N/A

**Software and algorithms**		

RNAseq analysis	GithHub	https://github.com/TsukudaS/HBV-XRN1
GraphPad	Prism	https://www.graphpad.com
BioRender	BioRender	https://help.biorender.com/hc/en-gb
ImageJ	Open-source software	https://doi.org/10.1038/nmeth.2089
Fiji (ImageJ)	Open-source software	https://doi.org/10.1038/nmeth.2019

**Other**		

Sequel II	PacBio	N/A
NovaSeq X Plus Sequencer	Novogene	N/A

### Experimental model and study participant details

#### Cell lines

HepG2-NTCP and Huh7.5 cells were maintained in Dulbecco’s Modified Eagles Medium (DMEM) containing Glutamax supplemented with 10% Fetal Bovine Serum, 50 U/mL Penicillin/Streptomycin, and non-essential amino acids (all reagents from Thermo Fisher Scientific). Cells were maintained at 37°C and in 5% CO_2_ and 18% O_2_. All cell lines were routinely monitored for morphological consistency by phase-contrast microscope at each passage, including assessment of characteristic morphology, adherence, growth rate, and absence of contamination. Periodically, housekeeping gene expression was monitored for functional stability and HBV receptor expression, HBV susceptibility, and XRN1 expression were monitored for phenotypic stability of the cell lines. Mycoplasma contamination was tested by using a kit (LONZA) and confirmed for negativity in all cell lines. HepG2-NTCP (a male HepG2 cell line stably expressing NTCP gene) was kindly provided by Dr. Stephan Urban at University of Heidelberg and Huh7.5 (a male cell line derived from Huh7 cell line) was kindly provided by Dr. Peter Simmonds at University of Oxford.

#### HBV strains

Heparin purified HBV inoculum derived from HepAD38 cell line^67^ were kindly provided by Dr. Ulrike Protzer at Technical University of Munich.

### Method details

#### Materials

Adenosine 3′,5′-diphosphate (pAp) was purchased from Cambridge BioScience, T7 mScript Standard mRNA Production System V2 was purchased from CellScript, Actinomycin D was purchased from Sigma-Aldrich, 7-Methylguanosine 5′-triphosphate was purchased form Insight Biotech. Antibodies used in this study (and their supplier) are: EDC4 (FORTIS), DDX6 (FORTIS), HBsAg (Stratech), HBcAg (Dako), HBcAg (Beacle Inc.) XRN1 (Thermo Fisher Scientific), β-Actin (Sigma), HBsAg (Abcam), anti-mouse Immunoglobulin HRP (Dako), anti-rabbit IgG HRP (Cytiva), anti-mouse IgG HRP (Abcam), anti-rabbit IgG (Alexa Fluor 488, 594 and 633, Thermo Fisher Scientific) and anti-7-methylguanosine (m^7^G)-Cap (Caltag Medsystems Ltd). The XRN1 expression plasmid, pLNHA-C1-HsXRN1, was purchased from Addgene (plasmid #66596 ^68^). Alexa 647-labelled preS1_2-48_ was synthesised by Thermo Fisher Scientific.

#### XRN1 knockout cell lines

HepG2-XRN1 KO cells were generated using a CRISPR-Cas9 system. HepG2-NTCP cells were seeded in 6 well plates and the following day co-transfected with crRNAs targeting XRN1 (5′-AAUGCGAAACAACACCUCCGUUUUAGA GCUAUGCUGUUUUG-3′), tracrRNA and Cas9 for 24 h (TransIT-CRISPR system, Sigma). After 48 h the cells were trypsinised, diluted into 96-well plates, clonal lines expanded and screened for XRN1 expression by western blotting.

#### HBV infection

HepG2-NTCP cells were seeded on collagen coated plasticware or collagen-coated coverslips and cultured in DMEM containing 2.5% DMSO for 72 h. Cells were inoculated with HBV (MOI of 300 genome copies) in the presence of 4% PEG8000 and 2.5% DMSO for 16 h. Viral inoculum was removed, and cells washed three times with PBS. Infected cells were maintained with DMEM containing 2.5% DMSO in 5% CO_2_ and 18% O_2_, with subsequent treatments and experimental timelines as described.

#### DNA transfection

HepG2-NTCP cells were transfected with indicated plasmids using either polyethylenimine (PEI) or FuGENE HD Transfection Reagent (Promega), according to the manufacturer’s protocol.

#### Cytotoxicity

Cytotoxicity was determined using a Lactate dehydrogenase CytoTox 96 Non-Radioactive Cytotoxicity Assay (Promega), according to the manufacturer’s protocol.

#### PreS1-NTCP cell binding assay

HepG2-NTCP cells were incubated with or without an unlabeled preS1_2-48_ peptide at 37°C for 1 h, followed by 50 nM Alexa 647-preS1_2-48_ peptide at 37°C for 1 h as described previously.^69^ After washing, the cells were fixed with 4% paraformaldehyde in PBS for 30 min, washed three times with PBS, incubated with 3% bovine serum albumin (BSA) in PBS containing DAPI for 30 min, and washed with PBS. Images were captured using an EVOS M5000 cell imaging system and processed with ImageJ.

#### Immunofluorescence staining

The cells were washed twice with PBS and fixed with 4% formaldehyde in PBS for 30 min. After fixation, samples were washed three times with PBS, permeabilised with 0.25% Triton X-100 in PBS for 20 min and blocked in 3% BSA in PBS for 30 min at room temperature (R.T). Primary antibodies were diluted in 1% BSA in 0.1% Tween 20 containing PBS (PBS-T) and incubated with the cells at 4 °C overnight or at R.T. for 1 h. Cells were washed three times with PBS and two times with PBS-T. Secondary antibodies and DAPI were diluted to 1:500 and 1:5000 in 1% BSA/PBS-T and the cells incubated with the antibodies at R.T. for 1 h in the dark. Cells were then washed three times with PBS, two times with PBS-T, and finally two additional times with PBS. Coverslips were mounted on to glass slides with VECTASHIELD Antifade Mounting Medium (Thermo Fisher Scientific). Images were collected using EVOS M5000 Cell Imaging Systems (Thermo Fisher Scientific) or SpinSR SoRa cell imaging systems (Olympus) and processed in ImageJ (Fiji).

#### Quantitative cDNA PCR (RT-qPCR)

Total cellular RNA was extracted using an RNeasy kit (Qiagen) and treated with TURBO DNase-free (Thermo Fisher Scientific), according to the manufacturer’s instructions. RNA was reverse transcribed using a cDNA synthesis kit (PCR Biosystems) according to the manufacturer’s protocol (25 °C, 10 min; 42 °C, 15 min; 48 °C, 15 min; 85 °C, 10 min). Cellular DNA was extracted using a QIAamp DNA Micro Kit (Qiagen) according to the manufacturer’s instructions. Gene expression was quantified using a SyGreen Blue Mix (PCR Biosystems) using a qPCR program of 95°C, 2 min; 45 cycles of 95°C, 5 s; 60°C, 30 s cccDNA was quantified according to the protocol described previously.^70^ Briefly, extracted DNA was treated with 5U of T5 exonuclease (NEB) for 37°C for 30 min followed by heat inactivation at 95°C. Treated DNA samples were quantified using a SyGreen Blue Mix using a qPCR program of 95°C, 10 min; 50 cycles of 95°C, 15 s; 60°C, 5 s; 72°C, 45 s; 88°C, 2 s. Oligonucleotides are listed in Table S1. Changes in gene expression were calculated relative to the housekeeper gene, *β2-microglobulin* (ΔCt method).

#### HBeAg and HBsAg detection

Secreted HBeAg and HBsAg were quantified by ELISA (Autobio, China) according to the manufacturer’s protocol.

#### SDS-PAGE and western blotting

Samples were lysed in RIPA buffer (50 mM Tris (pH 8.0), 150 mM NaCl, 1% Nonidet P-40 (NP-40), 0.5% sodium deoxycholate, and 0.1% sodium dodecyl sulfate supplemented with cOmplete Protease Inhibitor Cocktail (Merck). 4*x* Laemmli reducing buffer was added to samples before heating at 95°C for 10 min. Proteins were separated on 8 or 12% polyacrylamide gels and transferred to activated 0.45 μm PVDF membranes (Amersham). Membranes were blocked in 5% skimmed milk and proteins detected using specific primary and HRP-conjugated secondary antibodies. Signals were realised using a SuperSignal West Pico chemiluminescent substrate kit (Pierce) and images collected on a G:Box mini (Syngene).

#### Extracellular HBV DNA quantification

Extracellular HBV DNA was quantified according to the protocol described previously.^71^ Briefly, culture supernatant was treated with DNase I (Thermo Fisher Scientific) at 37°C for 60 min, then treated with 2*x* lysis buffer (100 mM Tris-HCl (pH7.4), 50 mM KCl, 0.25% Triton X-100, and 40% glycerol) containing 1 mM EDTA. HBV DNA was amplified by qPCR using primers for HBV DNA and quantified against a DNA referent standard curve.

#### Detection of core-associated HBV DNA

HBV infected HepG2-NTCP cells were washed with PBS and lysed in 100 mM Tris-HCl, pH 8.0, 0.2% NP-40 and 150 mM NaCl with cOmplete Protease Inhibitor Cocktail (Merck). After incubation at R.T. for 5 min, lysates were centrifuged at 20,000 g for 1 min and core particles isolated from the supernatant as previously reported.^72^ Briefly, 6 μM MgCl_2_, 0.1 U/mL DNase I (Thermo Fisher Scientific) and 0.1 mg/mL RNase A (Thermo Fisher Scientific) were added to the supernatant and incubated at 37°C for 2 h. Samples were incubated at 55°C for 1 h in the presence of 10 mM EDTA, 100 mM NaCl, 0.2 mg/mL protease K (QIAGEN) and 0.1% sodium dodecyl sulfate. DNA was purified by phenol-chloroform extraction and pellets resuspended in water. HBV DNA was amplified by qPCR using primers specific for HBV rcDNA and quantified against a DNA referent standard curve.

#### Northern blotting

RNA samples (0.5–10 μg) were incubated at 65°C for 15 min and quenched on ice for 2 min in the presence of 1*X* MOPS buffer (20 mM MOPS [pH 7.0], 2 mM sodium acetate, 1 mM EDTA), 2.2 M formaldehyde, 50% formamide and 25 μg/mL of Ethidium Bromide. The RNA mixture was resolved in a 1% agarose gel containing 2.2 M formaldehyde. After electrophoresis 18 S and 28 S ribosomal RNA species were visualised under UV light to verify the amount of RNA loaded and to assess degradation. The gel was denatured in a solution containing 50 mM NaOH for 5 min and incubated in 20*X* SSC buffer for 40 min. RNAs were transferred to a nylon membrane by capillary transfer using 20*X* SSC buffer and fixed by UV crosslinking. To detect HBV RNA, membranes were hybridised overnight at 65°C with a digoxigenin-labeled DNA probe spanning the entire HBV genome and visualised using a luminescent DIG detection kit (Roche) as described previously, with images collected on a G:Box mini (Syngene).^70^

#### Long-read sequencing and mapping the HBV transcriptome

HBV specific oligonucleotide enrichment, long-read sequencing and analysis was performed as previously reported.^6^ Briefly, RNA was extracted from HepG2-NTCP WT and XRN1 KO cells infected with HBV for 6 days using an RNeasy kit in accordance with the manufacturer’s instructions (Qiagen). RNA concentration was determined using a QuBit fluorometer (Thermo Fisher Scientific) and 300 ng of total RNA reverse transcribed with barcoded PacBio sample indexes (IDT) using Iso-Seq Express amplification primers (SMART cDNA synthesis kit, Takara Bio). The quality and quantity of cDNA was assessed using a Bioanalyser 2100 and Nanodrop spectrophotometer (Agilent and Thermo Fisher Scientific respectively). A panel of 120 bp biotinylated oligonucleotides was employed to enrich the library for HBV cDNAs, as previously described (xGen Lockdown platform,IDT).^6^ 150–300 ng of total RNA was used as input for target amplification (Iso-Seq Express Template Preparation system, PacBio), SMART cDNA synthesis (Takara Bio), and HiFi PCR amplification (Kapa Biosystems). Samples were sequenced using a Sequel II instrument to generate a PacBio ‘Hifi’ library, demultiplexed and stored as fastq archives. Reads were mapped to a 3.5 Kb overlength HBV reference genome (genotype D3, ayw strain, GenBank accession no. X02496.1, linearised at 1525 - the first base of the longest preCore transcript, and terminating at 1923 - the last base of the PAS signal, TATAAA) using minimap2.^35^^,^^73^ HBV reads were assigned to previously reported TSS to identify canonical or unspliced transcripts^34^ and splice junctions enumerated to identify non-canonical RNAs.^35^ The complete sequence dataset is available as an SRA at NCBI (BioProject ID: PRJNA1227884).

#### Short read sequencing of the HepG2 transcriptome

RNA was extracted from DMSO arrested WT and XRN1 KO22 cells using an RNeasy kit with on-column DNase treatment in accordance with the manufacturer’s instructions (Qiagen). mRNA library was prepared by poly A enrichment and analyzed by 300 bp paired read sequencing on a NovaSeq X Plus Sequencer (Novogene). Differential sequence analysis was performed in Bioconductor using the EdgeR package, with standard thresholds for assessing the significance of the changes seen (Log2 fold change >1, Adjusted *p*-value <0.05). Gene ontology (GO) analysis and Reactome assessment were determined using ClusterProfiler packages enrichGO and enrichPathway, respectively. The RNA-seq data is available as an SRA at NCBI (BioProject ID: PRJNA1228332). The analysis pipeline scripts are available as a GithHub repository (https://github.com/TsukudaS/HBV-XRN1).

#### FISH HCR probe design

Candidate FISH probe sequences (Tables S2, S3, and S4) were designed using Hybridisation Chain Reaction (HCR) split probe designer v0.1.1 (https://github.com/jefflee1103/HCRv3_probe_design/tree/v0.1.1) with the following parameters: oligo length, 25 nt; spacing length between probe partners: 2 nt; minimum spacing length between probes: 5 nt; ΔG -75 to −50; GC 40–60% and Tm: 55°C–75°C. The following regions from the HBV (AY661792.1) reference sequence were used as target sequences: total RNA (nucleotides 159–1792) and pCpg RNA (nucleotides1849-2808). A BLAST screen of candidate probes was performed to ensure no off-target matches occurred. Oligonucleotides were purchased from Integrated DNA technologies. Signal amplification sequences for the HBV-total probes were coupled to hairpin B3-Alexa Fluor 546 and HBV-pCpg probes coupled to hairpin B1-Alexa Fluor 647. Meta-stable hairpins were purchased from Molecular Instruments.

#### *In situ* RNA hybridisation

Cells on coverslips were washed twice with PBS and fixed with 4% formaldehyde in PBS for 30 min. Coverslips were washed four times with PBS and stored in PBS containing 10 mM Ribonucleoside Vanadyl Complex (RVC) (NEB). Cells were permeabilised in PBS/0.1% Triton X-100 for 10 min at R.T. followed by washes in PBS and 2*X* SSC. Cells were incubated in hybridisation buffer (2× SSC, 10% formamide, 10% dextran sulfate) for 30 min at 37°C and with 16 nM HBV RNA probes overnight. Cells were washed twice in pre-warmed wash solution (2× SSC, 10% formamide) at 37°C for 15 min, twice in 5*X* SSC containing 0.1% Tween 20 at R.T. for 5 min, and in amplification buffer (2× SSC, 10% dextran sulfate) at R.T. for 30 min. HCR amplifier hairpins (Molecular Instruments) were incubated at 95°C for 90 s and cooled to R.T. for 30 min before use. HCR amplification was carried out in an amplification buffer containing 0.06 μM HCR amplifier hairpins at R.T. for 90 min. Samples were washed five times with 5*X* SSC containing 0.1% Tween 20 at R.T. for 5 min, and once with PBS containing 0.1% Tween 20 at R.T. for 5 min. Samples were incubated in blocking buffer (1% BSA treated with RNAsecure, (Thermo Fisher Scientific), 0.1% Tween 20, 10 mM RVC) at R.T. for 30 min and incubated with primary antibody overnight at 4°C. Cells were washed three times with PBS containing 0.1% Tween 20 at R.T. for 5 min and treated with secondary antibody in blocking buffer at R.T. for 1 h. Cells were washed three times with PBS/0.1% Tween 20/DAPI (1 μg/mL) at R.T. for 5 min followed by PBS 0.1%/Tween 20 at R.T. for 5 min, treated with a 1 in 80 dilution of TrueBlack Plus (Thermo Fisher Scientific) for 5 min at R.T., rinsed twice with PBS at R.T. for 5 min and mounted using SlowFade Diamond mountant (Thermo Fisher Scientific). For the DNase and RNase digestion control experiments, DNase I (1 U/μL) or RNase T1 (1000 U/μL), RNase A (10 U/μL) and RNase III (1 U/μL) were used. Permeabilised cells were treated with DNase or RNases in PBS and incubated at 37°C for 1 h, washed three times with PBS, and probes hybridised. Images were captured on a IXplore IX83 SpinSR Super-Resolution Microscope System (Olympus) with a Yokogawa CSU-W1 SoRa spinning disc unit and Hammamatsu ORCA Fusion BT camera with Photometrics Prime 95b detection. Excitation was set with diode lasers at 405 nm, 488 nm, 561 nm and 647 nm and filters set for DAPI/Alexa 488/Alexa 568/Alexa 647. Images were captured with 60×1.4NA Oil objective as 0.2 μm z-slices and processed using ImageJ (Fiji).

#### Quantification of capped HBV RNA

Total cellular RNA was extracted using an RNeasy (Qiagen) kit, and a TURBO DNA-free Kit (Thermo Fisher Scientific) treated RNA was further purified using an RNeasy kit (Qiagen). 2 μg of RNA was incubated overnight at 4°C with protein A agarose beads treated with Rabbit IgG or anti-m^7^G monoclonal antibody in RIP buffer (Tris-HCl (pH 8.0), 150 mM NaCl, 0.1% NP-40, 1 mM EDTA) supplemented with RNase inhibitor (Promega). Beads were washed 5 times with RIP buffer and bound RNA eluted with 5 mM m^7^G sodium salt. Eluted RNA was purified using a Qiagen RNA extraction kit and quantified by RT-qPCR. Quantities of HBV pgRNA and total HBV RNAs were calculated relative to input total RNA.

#### Quantification of P-bodies

The P-body components EDC4 and DDX6 along with nuclei were imaged using an EVOS M5000 Cell Imaging Systems (Thermo Fisher Scientific). Quantification of EDC4, DDX6 puncta was performed with ImageJ software (Fiji) using the analyze Particles command after adjusting the threshold of images for 3 images per one condition from an experimental. This process was performed for datasets from 3 independent experiments. The analysis pipeline scripts are available as a GithHub repository (https://github.com/TsukudaS/HBV-XRN1).

#### Quantification of fluorescence and co-localised signals

Quantification of fluorescence signals and co-localised fluorescence signals were analyzed by using a Zeiss Arivis pipeline. To assess quantification and co-localisation, threshold signals for HCR viral RNA, EDC4 and DDX6 were adjusted by comparing mock and HBV infected cells. All images were processed by an automated workflow where thresholds were set, all slices in one image were overlaid to construct a 3D image and overlapping signals quantified.

#### *In vitro* HBV pgRNA synthesis

*In vitro* transcription of HBV RNA from plasmid templates was performed as described previously, with minor modifications.^39^ Briefly, pUC59-pgRNA^40^ plasmid was linearised by AseI digestion at 37°C for 1 h. The reaction was terminated by adding 1/20 volume of EDTA and DNA purified using a PCR Purification Kit (Qiagen). *In vitro* pgRNA transcription was performed in a 20 μL final volume using a T7 mScript standard mRNA production system by following the protocol provided by the manufacturer (CellScript), the DNA template was digested with 10 μL of a DNase cocktail containing 10 U of DNase I (NEB), 3 μL 10*X* DNase I buffer, 2 μL nuclease-free water and incubated at 37°C for 20 min. After purification of the RNA using RNeasy kit (Qiagen), capping and polyadenylation were performed following Cap1 and Cap0 mRNA protocol described in the user’s manual, and the RNA was again purified using RNeasy kit.

#### Cell-free assay to measure XRN1 digestion of synthetic pgRNA

4 μg of capped or monophosphorylated RNA was prepared in a 40 μL final volume containing 1*X* NEB buffer 3 (NEB), 1 U of RNase inhibitor (Promega), the reaction was dispensed into 4 tubes. 1 U of XRN1 enzyme (NEB) was added to each tube and incubated at 37°C for the times indicated. The reaction was terminated by adding 5 μL of 50 mM EDTA, and RNA purified using an Oligo Clean & Concentrator (Zymo Research). 300 ng of the purified RNA was evaluated using 1% non-denaturing TBE gel pre-stained SYBR Green II RNA Gel Stain (Thermo Fisher Scientific) and northern blotting with HBV specific probe.

#### RNA transfection

Huh7.5 cells were seeded at 5 × 10^4^ cells/well in 24 well-plates one day before transfection. The medium was changed to 400 μL of DMEM containing 1.5% FBS, 0.1 mM NEAA and 1 mM HEPES per well just before transfection, 100 ng IVT pgRNA mixed with 0.5 μL of Lipofectamine 2000 (Fisher Scientific) in 100 μL Opti-MEM reduced medium (Fisher Scientific) was added to cells, and spinoculated by centrifugation at 1000*x* g for 30 min at 37°C. Six hours later, the medium was replaced with DMEM containing 3% FBS, 0.1 mM NEAA and 1 mM HEPES. Medium was replaced every two days and samples were collected at 2-, 4- and 6-day post-transfection. HEK 293 cells were seeded at 5 × 10^4^ cells/well in 24 well-plates one day before transfection and transfected 25 ng RNA by spinoculated by centrifugation at 1000*x* g for 30 min at 37°C. One hour later, the medium was replaced with DMEM containing 10% FBS, 0.1 mM NEAA and 1 mM HEPES and cells were harvested at 1, 3, 5 and 9 h post transfection for RNA extraction.

### Quantification and statistical analysis

All experiments were repeated at least three times. All data are presented as mean values ±SEM. *p*-values were determined using a Mann–Whitney test (two group comparisons) or a Kruskal–Wallis ANOVA (multi group comparisons) using GraphPad Prism version 10 (GraphPad, San Diego, CA, (USA). In the figures ∗*p* < 0.05, ∗∗*p* < 0.01, ∗∗∗*p* < 0.001, ∗∗∗∗*p* < 0.0001, ns. denotes non-significant.
