# Supplementary material for: A key role for the exoribonuclease XRN1 in regulating the hepatitis B viral transcriptome
Source: iScience. 2026 Jun 11;29(7):116328. doi: 10.1016/j.isci.2026.116328 (PMC13276156; doi:10.1016/j.isci.2026.116328)
Supplement: Document S1. Figures S1–S7 and Tables S1–S4 [file mmc1.pdf]

iScience, Volume *29*

Supplemental information

A key role for the exoribonuclease XRN1 in regulating the hepatitis B viral transcriptome

Senko Tsukuda, Nadina Wand, James M. Harris, Olivia Dobrica, Medhi Boutasbih, Xiaxuan Zhu, Peter A.C. Wing, Peter Balfe, and Jane A. McKeating

Supplemental figures

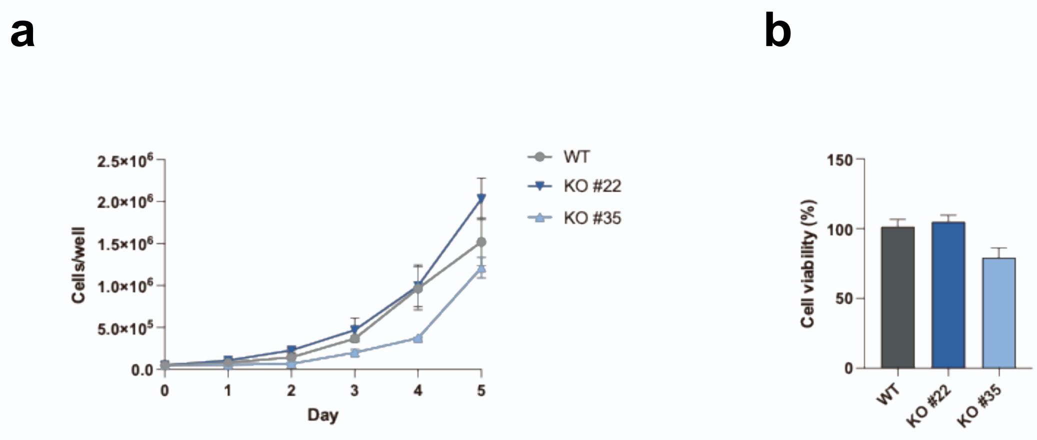

Figure S1. Cellular proliferation of XRN1 knockout cells.
(**a**) HepG2-NTCP WT or XRN1 KO cells were seeded in 24 well plates and the cell number counted at the indicated time points. (mean ± SD, n=6 from 3 independent experiments). (**b**) Cell viability after 9 days in culture with 2.5% DMSO was determined using a Lactate Dehydrogenase assay (Cytotox 96, Promega), with % viability relative to 3 days shown (n=6 from 3 independent experiments, one-way Kruskal-Wallis ANOVA with multiple comparisons). Please see also Fig.1.

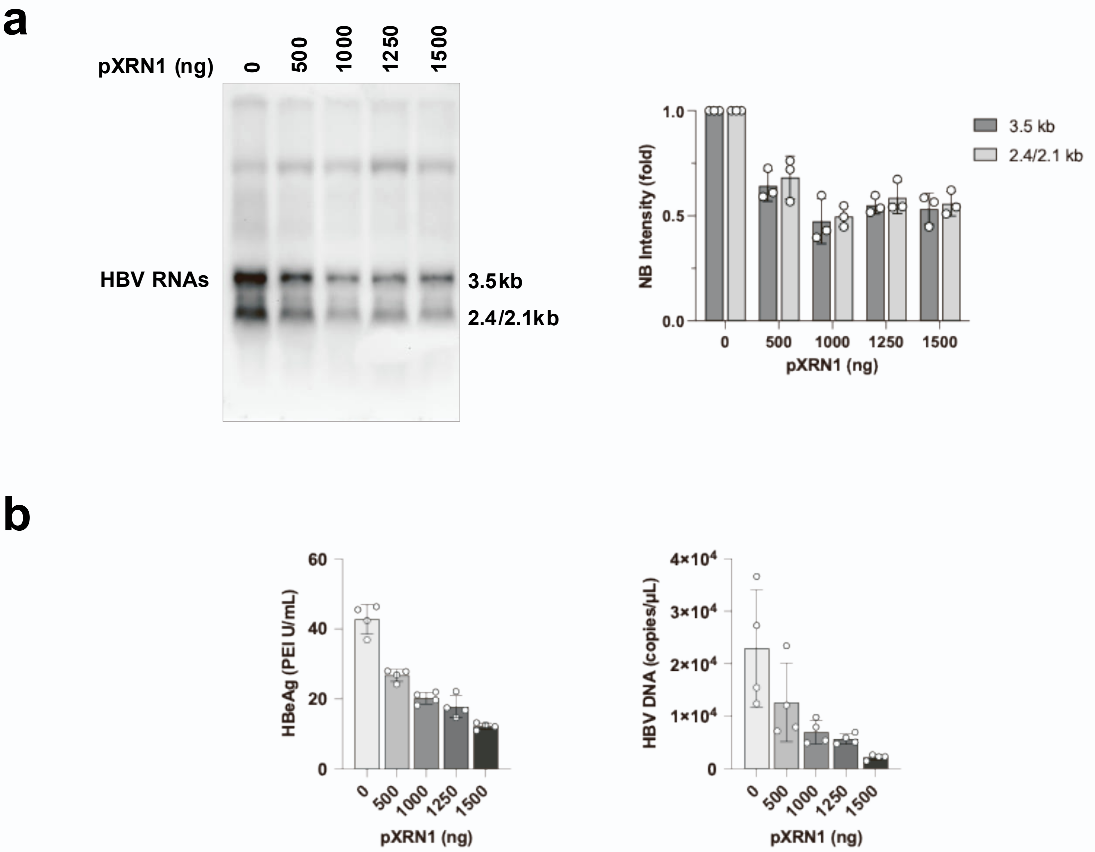

Figure S2. Northern blot analysis of HBV RNA.
(**a**) Uncropped image of northern blot shown in Fig.2a. The relative intensities of the 3.5 kb and 2.4/2.1 kb bands were quantified, with signals shown relative to the '0 ng' band. Data were obtained from 3 independent experiments. (**b**) Secreted HBeAg and HBV DNA were quantified by ELISA and RT-qPCR, respectively (mean ± SD, n=4 from 2 independent experiments). Please see also Fig.2.

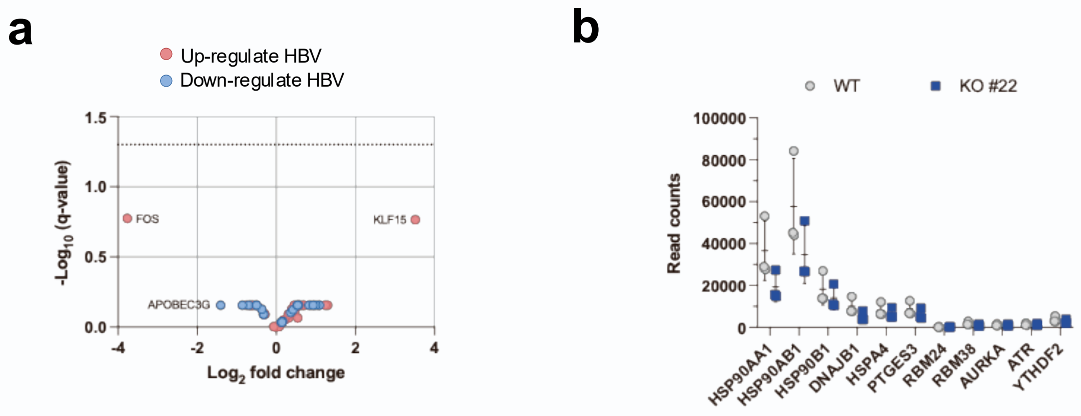

Figure S3. Gene expression of HBV associated host factors in WT and XRN1 KO cells.
(**a**) Differential expression of host factors reported to associate with up-regulation (red dots) or down-regulation (blue dots) of HBV transcription(Van Damme et al., 2021) in WT and XRN1 KO22 cells. The dotted line shows the threshold for significance, defined as 5%. (**b**) Read counts of the most abundant host factors are shown and data presented as the mean ± S.D. of 3 independent samples. Please see also Fig.2

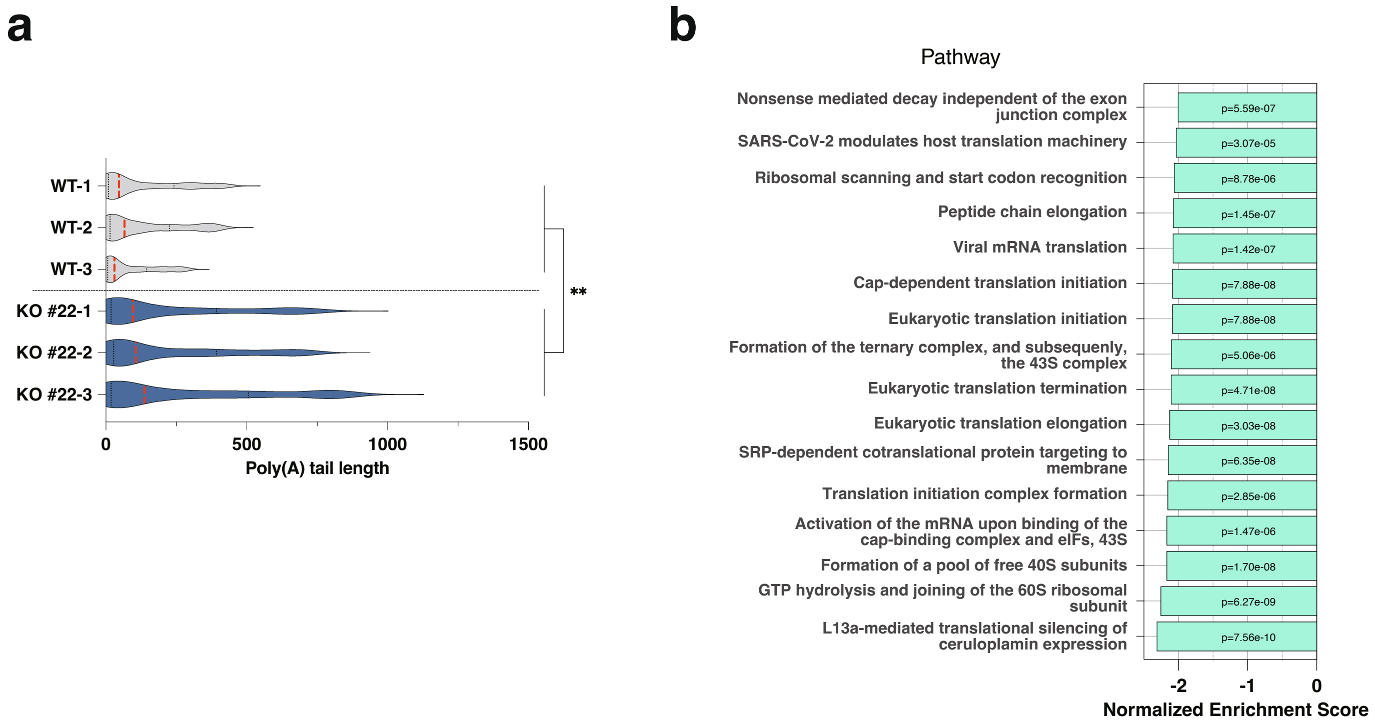

Figure S4. Poly(A) tail length of HBV transcripts.
(**a**) Poly(A) tail length of HBV transcripts was measured and statistical significance assessed using Wilcoxon matched-pairs signed rank test, ** $p < 0.01$. (**b**) GSEA Reactome analysis. Short-read RNA-seq of WT and XRN1 KO22 cells identified downregulated pathways in the KO22 cells and their normalised enrichment scores with adjusted p-values shown. Please see also Fig.3 Cellular proliferation of XRN1 knockout cells.

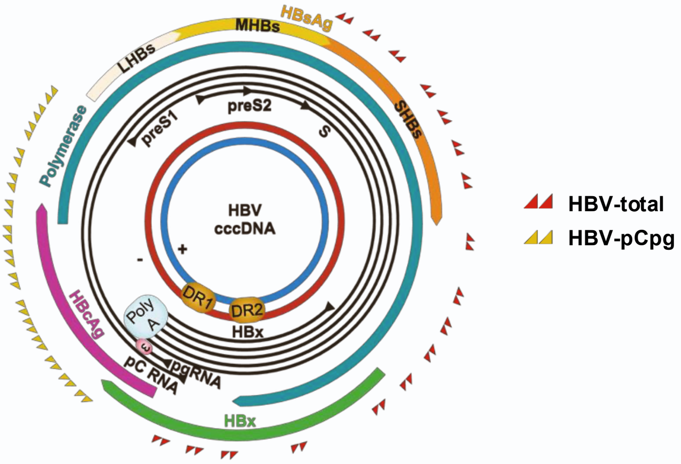

Figure S5. HBV HCR probe locations.
Schematic showing the location of HCR probes targeting total RNA (HBV-total, red triangles) or pCpgRNA (HBV-pCpg, yellow triangles) on the HBV genome. Please see also Fig.4.

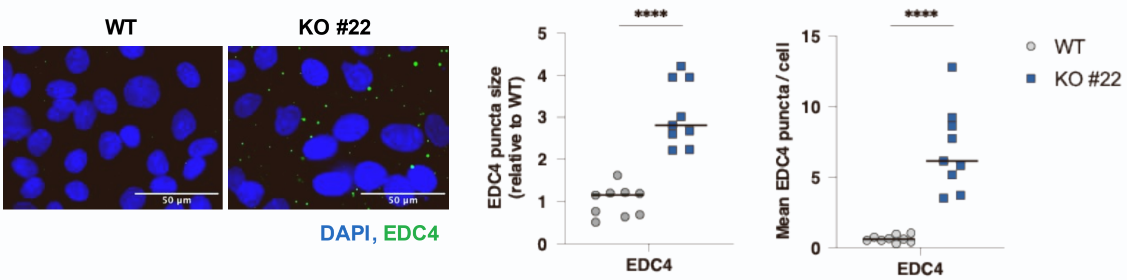

Figure S6. Imaging EDC4 puncta.
The P-body component EDC4 was imaged in HepG2-NTCP WT and KO22 cells with nuclei detected by DAPI staining (scale bar, 50µm) (9 images from 3 independent experiments). Mean EDC4 values are expressed relative to the number of nuclei per image, with statistical significance assessed using a Mann-Whitney test with correction for multiple comparisons (***$p < 0.001$, * $p < 0.05$). Please see also Fig.4.

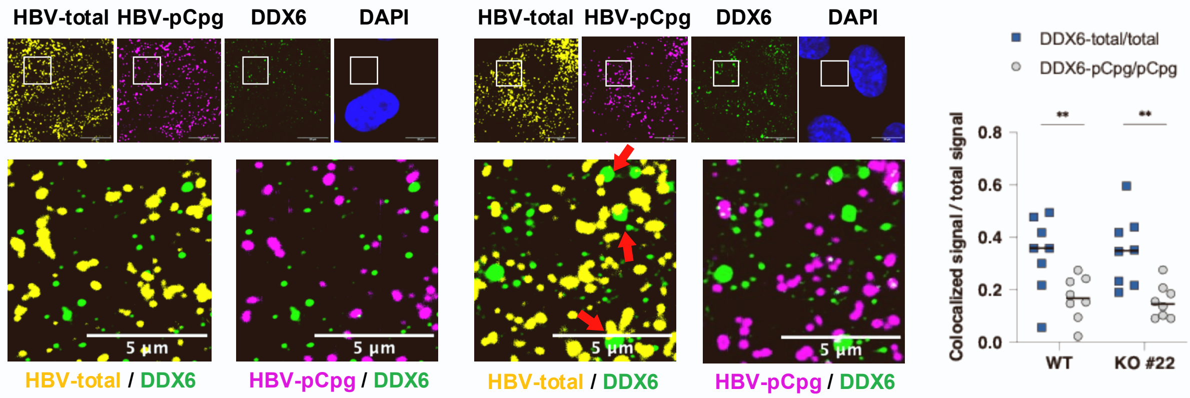

Figure S7. Colocalisation of HBV RNAs with DDX6 puncta.
HBV infected HepG2-NTCP WT or KO22 cells (6 dpi) were imaged for HBV-total (yellow), HBV-pCpg (magenta), DDX6 (green) and nuclear DAPI (blue), with scale bars of 10µm (main image) and 5µm (inset). Red arrows highlight colocalised HBV RNA-DDX6 large puncta that were a notable feature of the KO22 cells. The frequency of HBV-total or HBV-pCpg RNA puncta colocalising with DDX6 were quantified from 8 images collected from two independent infections and the average expressed relative to the overall viral signal with significance assessed using Mann-Whitney tests (** $p < 0.01$). Please see also Fig.4.

Supplemental tables

Table S1. qPCR primer sequences.

Name		Sequences
**B2M**	Forward	CTACACTGAATTCACCCCCACTG
	Reverse	ACCTCCATGATGCTGCTTACATG
**pCpgRNA**	Forward	GGGGAACTAATGACTCTAGCTACC
	Reverse	TTTAGGCCCATATTAGTGTTGACA
**total HBV RNAs**	Forward	ACGGGGCGCACCTCTCTTTA
	Reverse	GTGAAGCGAAGTGCACACGG
**HBV DNA**	Forward	AACACATAGCGCCTCATTTTG
	Reverse	AGGATTGCTGGTGGAAAGATT
**cccDNA**	Forward	GCCTATTGATTGGAAAGTATGT
	Reverse	AGCTGAGGCGGTATCTA
**PrP**	Forward	TGCTGGGAAGTGCCATGAG
	Reverse	CGGTGCATGTTTTCACGATAGTA

Table S2. HCR probes for HBV-pCpg.

Probe ID	Probe + Amplifier sequence
**HBV_D_pgRNA_HCRB1_01**	TGGAGGCTTGAACAGTAGGACATGATAGAAGAGTCTTCCTTTACG
**HBV_D_pgRNA_HCRB1_03**	CGATACAGAGCTGAGGCGGTATCTATAGAAGAGTCTTCCTTTACG
**HBV_D_pgRNA_HCRB1_05**	CAAAGAATTGCTTGCCTGAGTGCAGTAGAAGAGTCTTCCTTTACG
**HBV_D_pgRNA_HCRB1_07**	GCTGGATCTTCCAAATTAACACCCATAGAAGAGTCTTCCTTTACG
**HBV_D_pgRNA_HCRB1_09**	CCTGAACTTTAGGCCCATATTAGTGTAGAAGAGTCTTCCTTTACG
**HBV_D_pgRNA_HCRB1_11**	AGTGCGAATCCACACTCCGAAAGACTAGAAGAGTCTTCCTTTACG
**HBV_D_pgRNA_HCRB1_13**	AACAGTAGTCTCCGGAAGTGTTGATTAGAAGAGTCTTCCTTTACG
**HBV_D_pgRNA_HCRB1_15**	GAGACCTTCGTCTGCGAGGCGAGGGTAGAAGAGTCTTCCTTTACG
**HBV_D_pgRNA_HCRB1_17**	TATGAGTCCAAGGAATACTAACATTTAGAAGAGTCTTCCTTTACG
**HBV_D_pgRNA_HCRB1_19**	GAGGGTGTTTTCCAATGAGGGTTAATAGAAGAGTCTTCCTTTACG
**HBV_D_pgRNA_HCRB1_21**	GTAACTCCACAGTAGCTCCAAATTCTAGAAGAGTCTTCCTTTACG
**HBV_D_pgRNA_HCRB1_23**	TAACTGTGAGTGGGCCTACAAACTGTAGAAGAGTCTTCCTTTACG
**HBV_D_pgRNA_HCRB1_25**	TATCCAATGGCAAATATTTGGTAACTAGAAGAGTCTTCCTTTACG
**HBV_D_pgRNA_HCRB1_27**	GTGTCTAGTTTGGAAGTAATGATTATAGAAGAGTCTTCCTTTACG
**HBV_D_pgRNA_HCRB1_29**	GAGGCGCTATGTGTTGTTTCTCTCTTAGAAGAGTCTTCCTTTACG
**HBV_D_pgRNA_HCRB1_02**	GAGGAGGGCAGCAAACGGAAAAGAGATGATTAGGCAGAGGTGAAA
**HBV_D_pgRNA_HCRB1_04**	GAGGAGGGCAGCAAACGGAAAGATCTCGTACTGAAGGAAAGAAGT
**HBV_D_pgRNA_HCRB1_06**	GAGGAGGGCAGCAAACGGAATGGTGAGGTGAACAATGCTCAGGAG
**HBV_D_pgRNA_HCRB1_08**	GAGGAGGGCAGCAAACGGAACAGGTAGCTAGAGTCATTAGTTCCC
**HBV_D_pgRNA_HCRB1_10**	GAGGAGGGCAGCAAACGGAAGACATAACTGACTACTAGGTCTCTA
**HBV_D_pgRNA_HCRB1_12**	GAGGAGGGCAGCAAACGGAACAAATACTCTATAACTGTTTCTCTT
**HBV_D_pgRNA_HCRB1_14**	GAGGAGGGCAGCAAACGGAAGATAGGGGCATTTGGTGGTCTATAA
**HBV_D_pgRNA_HCRB1_16**	GAGGAGGGCAGCAAACGGAATTCTTCTTCTAGGGGACCTGCCTCG
**HBV_D_pgRNA_HCRB1_18**	GAGGAGGGCAGCAAACGGAAGATTCCCGAGATTGAGATCTTCTGC
**HBV_D_pgRNA_HCRB1_20**	GAGGAGGGCAGCAAACGGAAACAGGTACAGTAGAAGAATAAAGCC
**HBV_D_pgRNA_HCRB1_22**	GAGGAGGGCAGCAAACGGAATATAAGGGTCAATGTCCATGCCCCA
**HBV_D_pgRNA_HCRB1_24**	GAGGAGGGCAGCAAACGGAACACATTTTTTGATAATGTCTTGGTG
**HBV_D_pgRNA_HCRB1_26**	GAGGAGGGCAGCAAACGGAATTGGATAAAACCTAGCAGGCATAAT
**HBV_D_pgRNA_HCRB1_28**	GAGGAGGGCAGCAAACGGAATAGATGTTCTGGATAATAAGGTTTA
**HBV_D_pgRNA_HCRB1_30**	GAGGAGGGCAGCAAACGGAATATAATATACCCGCCTTCCATAGAG

Table S3. HCR probes for HBV-total.

Probe ID	Probe + Amplifier sequence
**HBV_D_total_RNA_HCRB3_01**	TTGGAGGACAGGAGGTTGGTGAGTGTTCCACTCAACTTTAACCCG
**HBV_D_total_RNA_HCRB3_03**	GAATTAGAGGACAAACGGGCAACATTTCCACTCAACTTTAACCCG
**HBV_D_total_RNA_HCRB3_05**	GATGTGATCTTGTGGCAATGACCCATTCCACTCAACTTTAACCCG
**HBV_D_total_RNA_HCRB3_07**	TATTGTTTACACAGAAAGGCCTTGTTTCCACTCAACTTTAACCCG
**HBV_D_total_RNA_HCRB3_09**	AAACAAGCGGCTAGGAGTTCCGCAGTTCCACTCAACTTTAACCCG
**HBV_D_total_RNA_HCRB3_11**	AAACGATGTATATTTGCGGGAGAGGTTCCACTCAACTTTAACCCG
**HBV_D_total_RNA_HCRB3_13**	TCTTTAAACAAACAGTCTTTGAAGTTTCCACTCAACTTTAACCCG
**HBV_D_total_RNA_HCRB3_15**	TCCCATAGGAATTTTCCGAAAGCCCTTCCACTCAACTTTAACCCG
**HBV_D_total_RNA_HCRB3_17**	CAAACAGTGGGGGAAAGCCCTACGATTCCACTCAACTTTAACCCG
**HBV_D_total_RNA_HCRB3_19**	GTTCCTTGAGCAGGAGTCGTGCAGGTTCCACTCAACTTTAACCCG
**HBV_D_total_RNA_HCRB3_21**	GCGCAGGATCCAGTTGGCAGCACAGTTCCACTCAACTTTAACCCG
**HBV_D_total_RNA_HCRB3_23**	CGTTCACGGTGGTCTCCATGCGACGTTCCACTCAACTTTAACCCG
**HBV_D_total_RNA_HCRB3_25**	CCCCGCCTGTAACACGAGCAGGGGTTTCCACTCAACTTTAACCCG
**HBV_D_total_RNA_HCRB3_27**	AGTTCCCCCTAGAAAATTGAGAGAATTCCACTCAACTTTAACCCG
**HBV_D_total_RNA_HCRB3_29**	CAGCCAGTGGGGGTTGCGTCAGCAATTCCACTCAACTTTAACCCG
**HBV_D_total_RNA_HCRB3_31**	GACGGGGAGTCCGCGTAAAGAGAGGTTCCACTCAACTTTAACCCG
**HBV_D_total_RNA_HCRB3_33**	ATGCCTACAGCCTCCTAGTACAAAGTTCCACTCAACTTTAACCCG
**HBV_D_total_RNA_HCRB3_02**	GTCCCTGCCTCTATATCTTTTGGAGGTTGGGGACTGCGAATTTTG
**HBV_D_total_RNA_HCRB3_04**	GTCCCTGCCTCTATATCTTTCTTGATAGTCCAGAAGAACCAACAA
**HBV_D_total_RNA_HCRB3_06**	GTCCCTGCCTCTATATCTTTACATCCAATGACATAGCCCATGAAA
**HBV_D_total_RNA_HCRB3_08**	GTCCCTGCCTCTATATCTTTGTTGGCGAGAAAGTGAAAGCCTGCT
**HBV_D_total_RNA_HCRB3_10**	GTCCCTGCCTCTATATCTTTTGGATCGGCAGAGGAGCCAGAAAGG
**HBV_D_total_RNA_HCRB3_12**	GTCCCTGCCTCTATATCTTTAACAGAGTTATCAGTCCCGAGAATG
**HBV_D_total_RNA_HCRB3_14**	GTCCCTGCCTCTATATCTTTGCCTCAAGGTCGGTCGTTGACATTG
**HBV_D_total_RNA_HCRB3_16**	GTCCCTGCCTCTATATCTTTGATGATGGGATGGGAATACAGGTGC
**HBV_D_total_RNA_HCRB3_18**	GTCCCTGCCTCTATATCTTTCACTGAACAAATGGCACTAGTAAAC
**HBV_D_total_RNA_HCRB3_20**	GTCCCTGCCTCTATATCTTTCTGCATGGTCCCGTGCTGGTGGTTG
**HBV_D_total_RNA_HCRB3_22**	GTCCCTGCCTCTATATCTTTCGACGGGACGTAAACAAAGGACGTC
**HBV_D_total_RNA_HCRB3_24**	GTCCCTGCCTCTATATCTTTGTAAGACCTTGGGCAAGAATTGGTG
**HBV_D_total_RNA_HCRB3_26**	GTCCCTGCCTCTATATCTTTTAGGAATCCTGATGTGATGTTCTCC
**HBV_D_total_RNA_HCRB3_28**	GTCCCTGCCTCTATATCTTTCCACCACGAGTCTAGACTCTGCGGT
**HBV_D_total_RNA_HCRB3_30**	GTCCCTGCCTCTATATCTTTACTTGGCACAGACCTGGCCGTTGCC
**HBV_D_total_RNA_HCRB3_32**	GTCCCTGCCTCTATATCTTTCGCCCCGTGGTCGGTCGGAACGGCA
**HBV_D_total_RNA_HCRB3_34**	GTCCCTGCCTCTATATCTTTCTTTAATCTAATCTCCTCCCCCAAC

Table S4. HCR probes for PPIB RNA.

Probe ID	Probe + Amplifier sequence
**Hsap_PPIB_HCRB5_01**	AAATTATCCACTGTTTTTGGAACAGAACTACCCTACAAATCCAAT
**Hsap_PPIB_HCRB5_03**	AGTTTGAAGTTCTCATCGGGGAAGCAACTACCCTACAAATCCAAT
**Hsap_PPIB_HCRB5_05**	TGTGGCGGACTACAGGGCCTGCACAAACTACCCTACAAATCCAAT
**Hsap_PPIB_HCRB5_07**	ATTCGTAGGTCAAAATACACCTTGAAACTACCCTACAAATCCAAT
**Hsap_PPIB_HCRB5_09**	GGCAGCAGCAGGAAGAAGACGGACCAACTACCCTACAAATCCAAT
**Hsap_PPIB_HCRB5_11**	AGGCGCAGCATCCACAGGCGGAGGCAACTACCCTACAAATCCAAT
**Hsap_PPIB_HCRB5_13**	GCTAGGGAGCCGGCGCCGCCACGCGAACTACCCTACAAATCCAAT
**Hsap_PPIB_HCRB5_15**	TCTCCGCCCTGGATCATGAAGTCCTAACTACCCTACAAATCCAAT
**Hsap_PPIB_HCRB5_17**	ATGAAGAACTGGGAGCCGTTGGTGTAACTACCCTACAAATCCAAT
**Hsap_PPIB_HCRB5_19**	ACCTCCATGCCCTCTAGAACTTTGCAACTACCCTACAAATCCAAT
**Hsap_PPIB_HCRB5_21**	CAGTCTGCGATGATCACATCCTTCAAACTACCCTACAAATCCAAT
**Hsap_PPIB_HCRB5_23**	GCCTGTGGAATGTGAGGGGAGTGGGAACTACCCTACAAATCCAAT
**Hsap_PPIB_HCRB5_02**	CTCACTCCCAATCTCTATAATTTCCGAAGAGACCAAAGATCACCC
**Hsap_PPIB_HCRB5_04**	CTCACTCCCAATCTCTATAATCACCGTAGATGCTCTTTCCTCCTG
**Hsap_PPIB_HCRB5_06**	CTCACTCCCAATCTCTATAACGGTCACTCAAAGAAAGATGTCCCT
**Hsap_PPIB_HCRB5_08**	CTCACTCCCAATCTCTATAAGTGACTTTGGGCCCCTTCTTCTTCT
**Hsap_PPIB_HCRB5_10**	CTCACTCCCAATCTCTATAAGCGATGAGGGCGGCGGCAAGGAGCA
**Hsap_PPIB_HCRB5_12**	CTCACTCCCAATCTCTATAAAAGCAGCCCGGACAGCTGAGGCCGG
**Hsap_PPIB_HCRB5_14**	CTCACTCCCAATCTCTATAAGGTGGGGGGGACTGGGGTTGCTCGC
**Hsap_PPIB_HCRB5_16**	CTCACTCCCAATCTCTATAAATTACACGATGGAATTTGCTGTTTT
**Hsap_PPIB_HCRB5_18**	CTCACTCCCAATCTCTATAATTGCCTGCGTTGGCCATGCTCACCC
**Hsap_PPIB_HCRB5_20**	CTCACTCCCAATCTCTATAAAACACCACATGCTTGCCATCTAGCC
**Hsap_PPIB_HCRB5_22**	CTCACTCCCAATCTCTATAAGGTTTATCCCGGCTGTCTGTCTTGG
**Hsap_PPIB_HCRB5_24**	CTCACTCCCAATCTCTATAACGCTCCACCAGATGCCAGCACCGGG
